# A quantitative metric of pioneer activity reveals that HNF4A has stronger in vivo pioneer activity than FOXA1

**DOI:** 10.1186/s13059-022-02792-x

**Published:** 2022-10-17

**Authors:** Jeffrey L. Hansen, Barak A. Cohen

**Affiliations:** 1 The Edison Family Center for Genome Sciences and Systems Biology, School of Medicine, Washington University in St. Louis, Saint Louis, MO USA; 2 Department of Genetics, School of Medicine, Washington University in St. Louis, Saint Louis, MO USA; 3 Medical Scientist Training Program, Washington University in St. Louis, St. Louis, MO USA

**Keywords:** Pioneer factor, Pioneer activity, Transcription factor binding, FOXA1, HNF4A, Genomics

## Abstract

**Background:**

We and others have suggested that pioneer activity — a transcription factor’s (TF’s) ability to bind and open inaccessible loci — is not a qualitative trait limited to a select class of pioneer TFs. We hypothesize that most TFs display pioneering activity that depends on the TF concentration and the motif content at their target loci.

**Results:**

Here, we present a quantitative in vivo measure of pioneer activity that captures the relative difference in a TF’s ability to bind accessible versus inaccessible DNA. The metric is based on experiments that use CUT&Tag to measure the binding of doxycycline-inducible TFs. For each location across the genome, we determine the concentration of doxycycline required for a TF to reach half-maximal occupancy; lower concentrations reflect higher affinity. We propose that the relative difference in a TF’s affinity between ATAC-seq labeled accessible and inaccessible binding sites is a measure of its pioneer activity. We estimate binding affinities at tens of thousands of genomic loci for the endodermal TFs FOXA1 and HNF4A and show that HNF4A has stronger pioneer activity than FOXA1. We show that both FOXA1 and HNF4A display higher binding affinity at inaccessible sites with more copies of their respective motifs. The quantitative analysis of binding suggests different modes of binding for FOXA1, including an anti-cooperative mode of binding at certain accessible loci.

**Conclusions:**

Our results suggest that relative binding affinities are reasonable measures of pioneer activity and support the model wherein most TFs have some degree of context-dependent pioneer activity.

**Supplementary Information:**

The online version contains supplementary material available at 10.1186/s13059-022-02792-x.

## Background

Activating silent genes requires transcription factors (TFs) to bind and open DNA when their motifs are occluded by nucleosomes. Activating silent genes is postulated to involve two qualitatively different classes of TFs, pioneer factors (PFs), and non-pioneer factors (nonPFs) [1, 2]. According to this hypothesis, PFs bind to nucleosome-occluded DNA and make it accessible to nonPFs, which then recruit the cofactors required to activate transcription. However, we recently showed that both a canonical PF, FOXA1, and a nonPF, HNF4A, can independently bind, open, and then activate nearby genes [3], and many TFs possess unique ways of binding and opening nucleosomal DNA [4–8]. From these data, we propose that most TFs have quantifiable pioneer activity that depends on their nuclear concentrations and the motif content at their target loci. Here, we present a metric that can quantify in vivo pioneer activity.

**Fig. 1:**
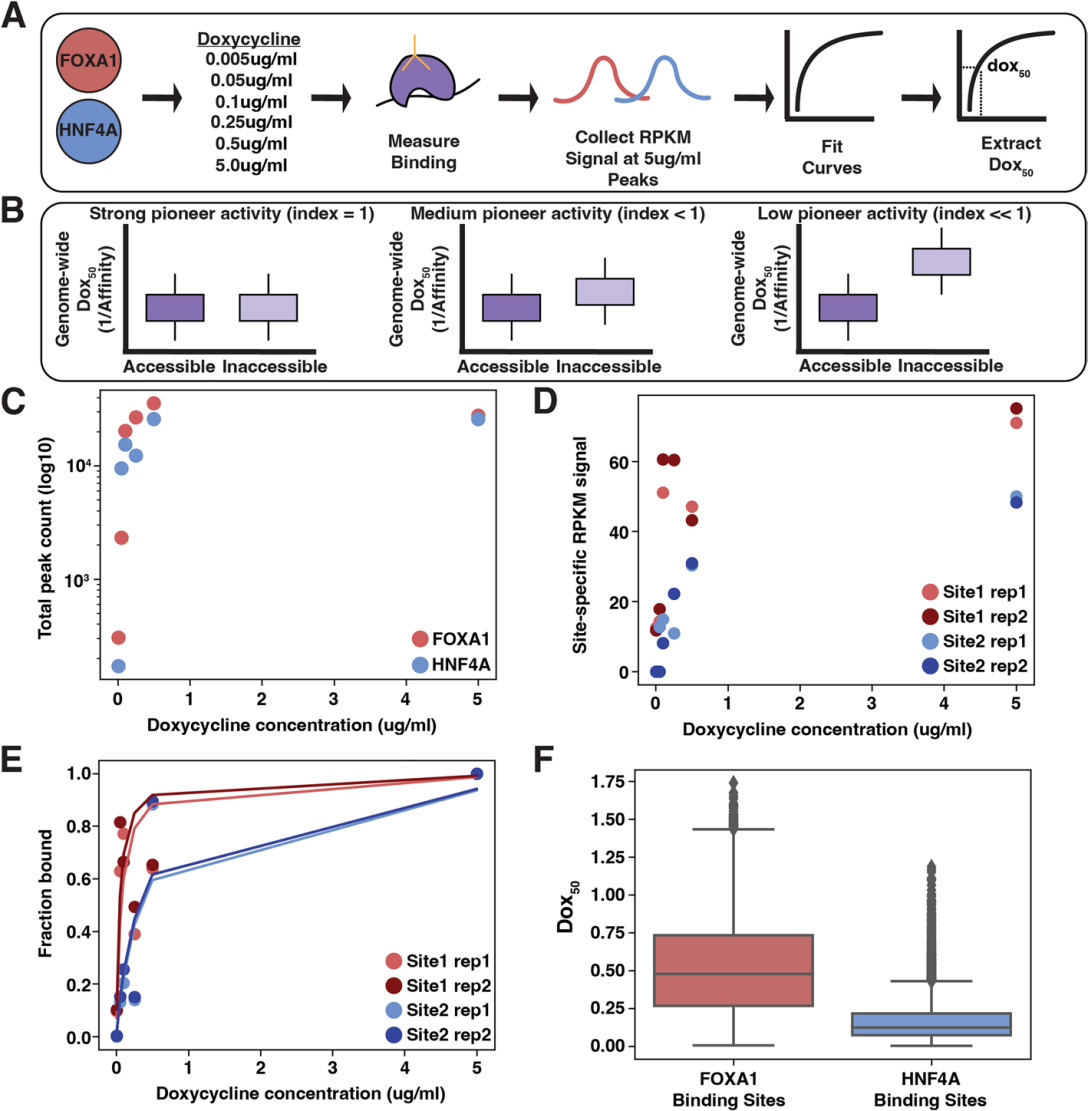
Experimental design to compute pioneer activity. **A** We induced FOXA1 or HNF4A across a 1000-fold dox range, measured binding, collected binding signals at a reproducible set of binding sites, and then extracted a dox_50_ for each site. **B** Maximal pioneer activity (index = 1) occurs when a TF’s average affinity in accessible regions is similar to that in inaccessible regions. Pioneer activity decreases (index < 1) as the affinity in inaccessible regions decreases, causing an increase in the dox_50_ measurement. **C** The number of total peaks for each TF across each dox induction level. **D** Replicate RPKM binding signal at two example genomic sites. **E** Replicate fitted lines at two example genomic sites. **F** Full distribution of dox_50_ values for each TF

A metric for pioneer activity should reflect the relative difference in binding affinity between accessible and inaccessible DNA, where strong pioneer activity is a result of a smaller difference in relative affinity. Normally, affinity is quantified by measurements of in vitro equilibrium dissociation constants, or K_d_s. We propose a method to estimate in vivo affinities in parallel by using our previously established doxycycline-inducible (dox-inducible) TF expression lines [3]. We expressed endodermal TFs FOXA1 and HNF4A across a 1000-fold range, measured binding and accessibility with CUT&Tag [9] and ATAC-seq [10], and then extracted the dox concentration at which each site was half-maximally bound, or the site’s “dox_50_” (Fig. 1A). Each site’s dox_50_ reflected the TF’s affinity for that site. The ratio of the average dox_50_ at accessible versus inaccessible binding sites is the TF’s pioneer activity index. We show that HNF4A has stronger overall pioneer activity and that both TFs can compensate for weaker affinity at inaccessible binding sites when there are more copies of their motifs. The distribution of pioneer activities across the genome supports the hypothesis that most TFs have pioneer activity given sufficient TF levels and motif content.

## Results

### A quantitative metric for pioneer activity

An appropriate measure of pioneer activity should capture the relative difference of TF binding between accessible and inaccessible sites in the genome. In principle, we could compare the dissociation constant (K_d_) of a TF at accessible and inaccessible sites as a measure of pioneer activity, since the K_d_ is the concentration of TF required to reach half-maximal binding. In practice, computing an absolute K_d_ inside cells is impractical. While it is possible to measure apparent K_d_s using fluorescently-labeled TFs, the throughput and resolution required to make accurate genome-wide calculations is impractical. We propose a related measure that uses doxycycline-inducible (dox-inducible) TFs to compute the dox_50_, the dox concentration required to reach half-maximal binding inside cells. By inducing TF levels over a wide range of dox concentrations and measuring the resulting binding, we determine a dox_50_ for every location in the genome in parallel. This method assumes linearity of expression as a function of doxycycline concentration, and we have shown previously that the expression of targets of our inducible TFs increases linearly with dox concentrations [3].

The ratio of the average dox_50_ at accessible versus inaccessible sites is a TF’s “pioneer activity index.” A TF with maximal pioneer activity will bind with equal affinity to accessible and inaccessible DNA and have a pioneer activity index of 1. Decreased affinity (higher dox_50_) at inaccessible sites will lower the index towards 0 (Fig. 1B). Because the measurements at accessible and inaccessible sites are made at the same time in the same nucleus, the dox concentrations (or TF concentrations) cancel out, allowing us to compare pioneer activity indices of different TFs to each other [11]. This strategy allows us to circumvent the challenge of measuring effective nuclear TF concentration while maintaining the physiological relevance of our in vivo pioneer activity measurements.

### Measurement of dox_50_ for FOXA1 and HNF4A

FOXA1 and HNF4A are liver TFs that are commonly used to reprogram embryonic fibroblasts to endoderm progenitor cells [12, 13]. FOXA1 is a canonical PF and HNF4A a nonPF, and the two are suggested to work in a collaborative and sequential fashion to activate their target genes [1, 14]. We previously tested FOXA1 and HNF4A’s behavior in an ectopic setting by expressing them within K562 blood cells, a lineage in which neither TF is expressed and that should present the TFs with unique complements of chromatin and cofactors. We created clonal K562 lines that expressed either inducible FOXA1 or HNF4A and showed that both TFs could independently bind and open inaccessible chromatin and activate nearby genes [3].

Based on the ability of FOXA1 and HNF4A to independently bind, open, and activate in an ectopic cell line, we expected both TFs would have similar pioneer activity. To test this prediction we attempted to measure each TF’s pioneer activity index using the same dox-inducible FOXA1 or HNF4A K562 lines (Fig. 1A). We first treated each TF line with a 1000-fold range of dox (0.005, 0.05, 0.25, 0.1, 0.5, and 5.0μg/ml) and measured resultant binding. Read normalized binding signal (RPKM) was highly correlated between replicates (Fig. S1). We found that each TF transitioned from binding hundreds of sites to tens of thousands of sites in a similar fashion as we increased the dox concentration and that each TF increased similarly, suggesting that neither TF was operating exclusively at either end of its dynamic range (Fig. 1C). We then collected the overlapping set of binding sites between each TF’s replicates in the 5.0μg/ml sample and at each site plotted the read normalized signal (RPKM) from the other induction levels (Fig. 1D). Generally we observe that at each site, a TF’s binding signal saturates in an expected fashion as its expression increases; we refer to this type of binding as the “saturation modality” (Fig. S2). We then fit Eq. 1 to these distributions.

1 $$\textrm{Fraction}\ \textrm{bound}=\frac{1}{1+\frac{{\textrm{dox}}_{50}}{\left[\textrm{dox}\right]}}$$

In order to fit Eq. 1, we normalized each site’s RPKM signal to the signal in the 5.0-μg/ml sample to convert our measurements into fractional binding (Fig. 1E). We found that at some sites the binding signal peaked prior to the 5.0-μg/ml sample (fraction bound > 1 in any of the first five induction levels). We removed these sites to prevent poor fitting. This left us with 11,557 FOXA1 binding curves and 5940 HNF4A binding curves with highly similar fitted lines across replicates (Figs. 1E and S3). We extracted dox_50_ values from these lines and found similar results between replicates (Fig. S4) and so we averaged each site’s replicate dox_50_ value for the remaining analyses. The resulting distributions of each TF’s genome-wide dox_50_ values show that FOXA1 has a much larger variance in dox_50_ values than HNF4A (Fig. 1F), suggesting that FOXA1 binding generally depends more on the genomic environment than HNF4A.

### Measurement of pioneer activity indices for FOXA1 and HNF4A

The dox_50_ distributions in Fig. 1 suggest that HNF4A may bind more consistently across the genome but does not explicitly report on the difference in binding affinity between accessible and inaccessible sites. We therefore classified each site as either accessible or inaccessible based on ATAC-seq peaks collected in these cell lines before dox induction [3]. Seventeen percent of FOXA1’s binding sites occurred in accessible regions and 83% occurred in inaccessible regions (prior to the above filtering step: 36% accessible and 64% inaccessible). Thirty-six percent of HNF4A’s binding sites occurred in accessible regions and 64% occurred in inaccessible sites (prior to filtering, 49% accessible and 51% inaccessible). A larger proportion of FOXA1-accessible sites were filtered out due to the unexpected anti-cooperative binding modality that we identified and that we discuss further below.

**Fig. 2:**
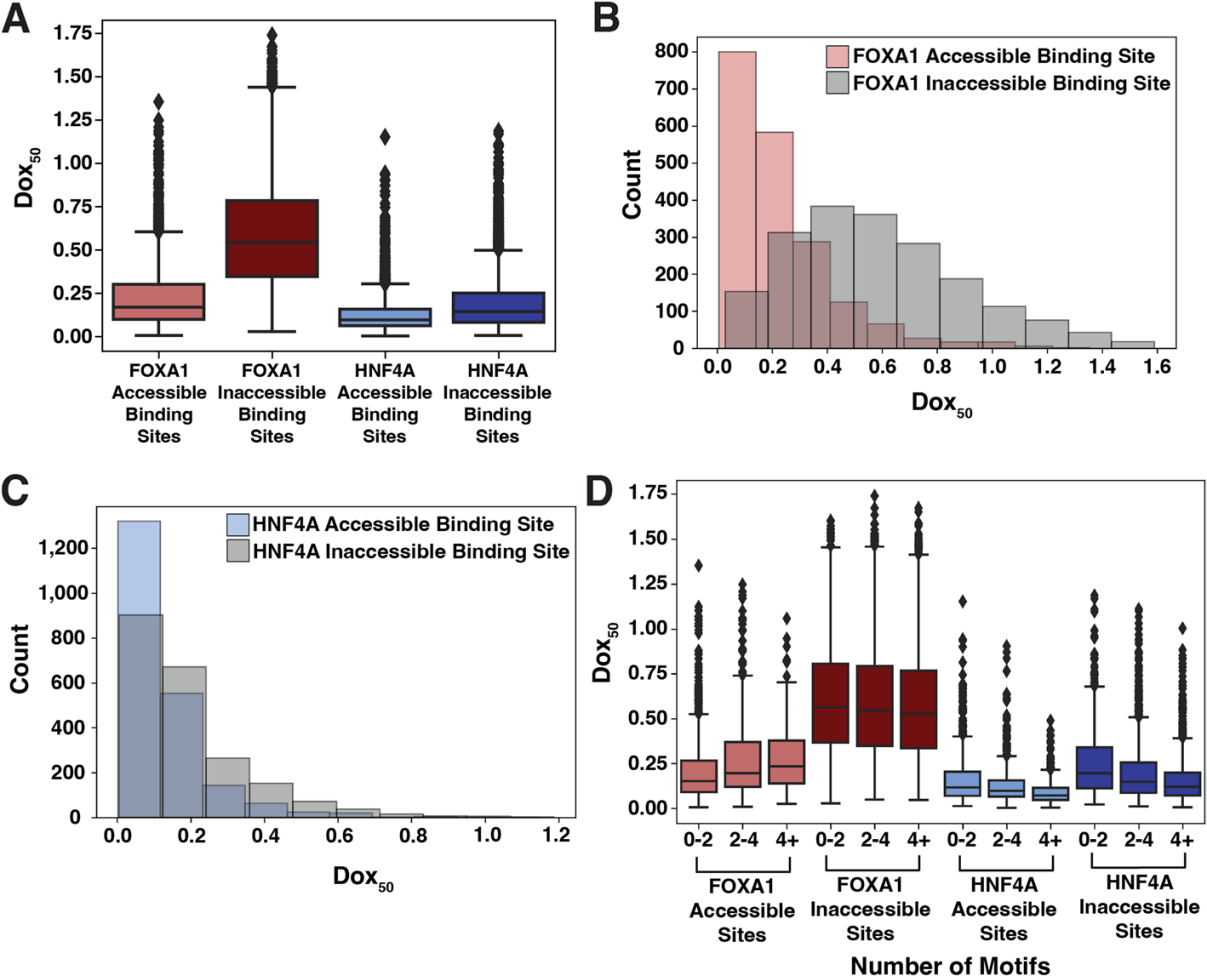
HNF4A has stronger pioneer activity than FOXA1. **A** Distributions of dox_50_ estimates extracted from binding curves at FOXA1-accessible binding sites (*n* = 1930), FOXA1 inaccessible binding sites (*n* = 9627), HNF4A accessible binding sites (*n* = 2135), and HNF4A inaccessible binding sites (*n* = 3805). **B**, **C** Distributions from FOXA1 (**B**) and HNF4A (**C**) shown in histogram form. **D** Same plot as **A** but each genomic binding site is binned by whether the site has < 2, ≥ 2 but < 4, or ≥ 4 motifs as called by FIMO (*p* = 1e−3)

Comparing the dox_50_ distributions between accessible and inaccessible sites revealed that the binding of HNF4A is less affected by inaccessible DNA than FOXA1 (Figs. 2A–C and S4). We then computed pioneer activity indices for each TF by dividing the average dox_50_ for accessible sites by the average dox_50_ for inaccessible sites. A TF with maximal pioneer activity would have equal affinity across accessible and inaccessible sites and therefore have an index of 1. Any reduction in affinity at inaccessible sites increases the average inaccessible dox_50_ and thus decreases the index. HNF4A’s index was 0.680 and FOXA1’s was 0.391, demonstrating that HNF4A has stronger global pioneer activity than FOXA1. HNF4A has stronger pioneer activity even when considering all sites without filtering (Fig. S5).

We next considered whether the motif content at each binding site affected pioneer activity. We subset each TF’s binding sites into those that had less than 2, between 2 and 4, or more than 4 motifs specific to the respective TF and re-plotted the dox_50_ distributions. For both FOXA1 and HNF4A, higher motif content at inaccessible binding sites correlated with lower dox_50_ distributions (Fig. 2D). Unexpectedly, FOXA1-accessible sites with more motifs had lower affinity. We speculate this may be driven by anti-cooperative binding behavior and explore this phenomenon further below. We conclude that HNF4A has stronger pioneer activity in K562 cells and that weak affinity binding at inaccessible regions can be compensated for by both TFs by the presence of additional motifs.

### Chromatin modifications explain some of the variance in dox_50_ values

We built a linear model (Eq. 2) to try to explain the variance in dox_50_s for FOXA1 and HNF4A where C(Accessibility) is each binding site’s accessibility prior to TF induction. Accessibility explained 17% of the variance in FOXA1’s dox_50_s but only 4% of HNF4A’s. While these data further underscore the greater role that accessibility plays on FOXA1 binding than HNF4A, they also reveal that most of the variance in dox_50_ values between genomic loci must be explained by some other variable.

2 $${\textrm{Dox}}_{50}\sim \textrm{C}\ \left(\textrm{Accessibility}\right)$$

**Fig. 3:**
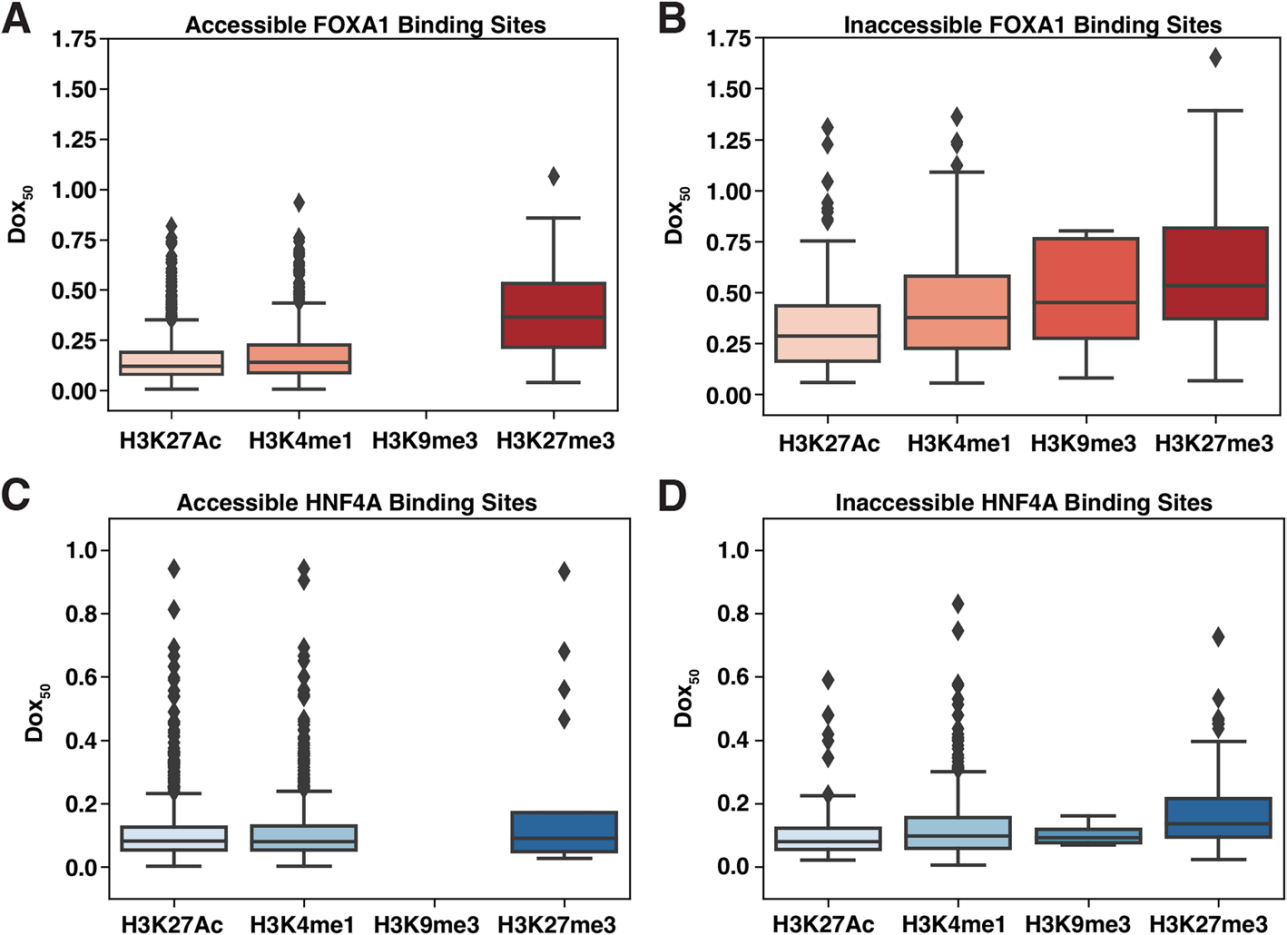
Dox_50_ distributions across different chromatin modifications. **A** Dox_50_ values for FOXA1-accessible binding sites that overlapped H3K27AC (*n =* 1288), H3K4me1 (*n =* 755), H3K9me3 (*n* = 0), and H3K27me3 (*n* = 21). **B** Dox_50_ values for FOXA1 inaccessible binding sites that overlapped H3K27AC (*n =* 203), H3K4me1 (*n =* 352), H3K9me3 (*n* = 12), and H3K27me3 (*n* = 277). **C** Dox_50_ values for HNF4A accessible binding sites that overlapped H3K27AC (*n =* 1147), H3K4me1 (*n =* 1111), H3K9me3 (*n* = 0), and H3K27me3 (*n* = 17). **D** Dox_50_ values for HNF4A inaccessible binding sites that overlapped H3K27AC (*n =* 140), H3K4me1 (*n =* 416), H3K9me3 (*n* = 4), and H3K27me3 (*n* = 135)

We hypothesized that some of the remaining variance may be explained by the different chromatin modifications present at different target loci and predicted that binding sites with active marks would have lower dox_50_ distributions (easier binding) and binding sites with silent marks would have higher dox_50_ distributions (harder binding). We further subset each TF’s accessible or inaccessible binding sites into those that overlap common K562 marks [15]. H3K4me1 marks enhancers [16], H3K27Ac marks activity [17], and H3K9me3 and H3K27me3 are two modifications shown previously to suppress pioneer activity [18]. The accessible sites overlapped much more often with active marks than silencing marks, and we found that no FOXA1 or HNF4A accessible sites were marked with H3K9me3 (Fig. 3).

As predicted, FOXA1 or HNF4A binding sites that overlapped H3K27Ac or H3K4me1 chromatin modifications had lower dox_50_ distributions than those that overlapped H3K9me3 or H3K27me3 (Fig. 3). These effects were present even after we subset binding sites by accessibility, suggesting that the chromatin modifications can affect binding in ways that are separable from the effects of accessibility. However, when we individually added each chromatin modification (plus an interaction term) to the model in Eq. 1, we found that accounting for these marks did not have large effects on the ability of the model to predict dox_50_ values for either TF. H3K27ac levels explained 2% of FOXA1’s dox_50_ variance, H3K4me1 explained 1%, and H3K27me3 explained <1%. For HNF4A, H3K27ac explained 2%, H3K4me1 explained 2%, and H3K27me3 explained <1%. All interaction terms were negligible. We also considered whether a site’s DNA methylation level [19] correlated with its dox_50_ value but only identified a minor inhibition of binding exclusively at accessible FOXA1 binding sites (Fig. S6). Together these data suggest that something besides the epigenetic landscape of loci is having a large effect on the pioneering activity of TFs.

### FOXA1 behaves anti-cooperatively at a subset of accessible binding sites

**Fig. 4:**
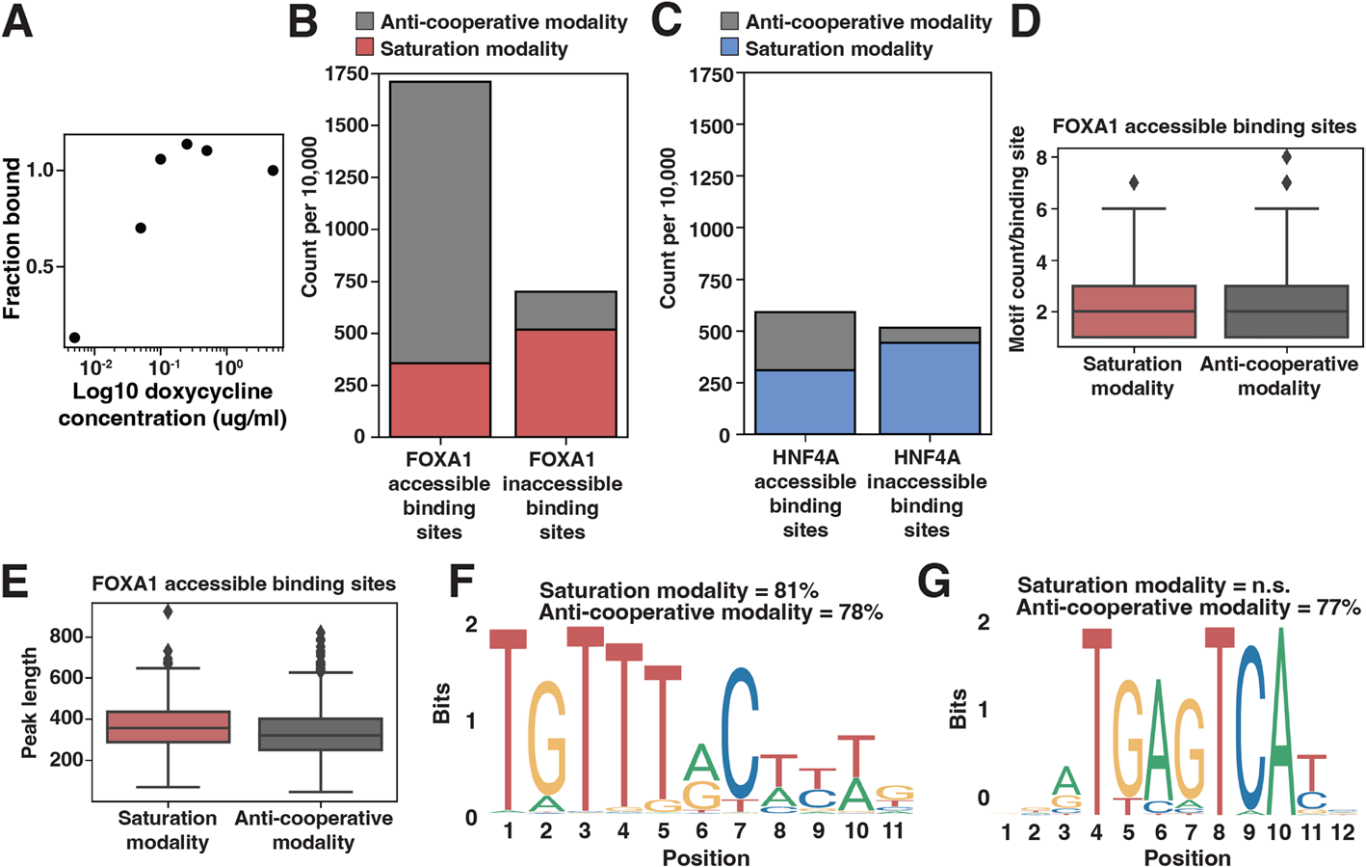
Characterization of anti-cooperative binding behavior. **A** Example binding curve at a single genomic site that exhibits anti-cooperative behavior. **B**, **C** A sample of 10,000 FOXA1 (**B**) or HNF4A (**C**) accessible (left bar) or inaccessible (right bar) binding sites colored by if they display saturation binding behavior (red or blue) or anti-cooperative binding behavior (gray). **D** FOXA1 motif count between the accessible binding sites from **B** that display either saturation or anti-cooperative binding behavior. Motifs were called from FIMO with a *p*-value threshold of 1e−3. **E** Binding peak length between the accessible binding sites from **B** that display either saturation or anti-cooperative binding behavior. **F** The most enriched motif discovered in FOXA1-accessible saturation and anti-cooperative peaks was FOXA1 (JASPAR MA0148.1). It is significantly enriched for both the saturation behavior (*p* = 2.19e−001) and anti-cooperative behavior (*p* = 1.23e−01). **G** The second most enriched motif discovered in FOXA1-accessible anti-cooperative peaks was AP1 (JASPAR MA1141.1). It was not discovered in the saturation behavior peaks. It is significantly enriched for only the anti-cooperative behavior (*p* = 1e−008)

While examining individual binding sites and their fitted curves, we observed a repeating pattern at a subset of genomic locations where the binding signal increased to a peak at the third (0.1μg/ml) or fourth (0.25μg/ml) induction level and then decreased, suggesting anti-cooperative behavior (Figs. 4A and S7). To quantify the prevalence of this “anti-cooperative modality” we sampled 10,000 peaks from the original set of unfiltered FOXA1 or HNF4A accessible or inaccessible binding sites and then counted how many displayed saturation behavior (peak at 5μg/ml, Fig. S2) and how many displayed anti-cooperative behavior (peak at 0.1μg/ml or 0.25μg/ml, Figs. 4A and S7). We drew strict criteria for the saturation behavior (binding signal must increase sequentially until maximum at 5μg/ml) and for the anti-cooperative behavior (binding signal must increase sequentially until peak and then decrease sequentially) such that not every one of the 10,000 sampled binding sites were included within one of these two modalities. We found that the anti-cooperative behavior occurs most often at accessible FOXA1 binding sites (Fig. 4B–C). Anti-cooperative behavior does not appear to depend on the number of motifs at each peak (Fig. 4D) or the length of each peak (Fig. 4E).

We considered whether another TF might be contributing to anti-cooperative behavior by searching for enriched motifs in either saturation-type accessible FOXA1 binding sites or anti-cooperative-type sites. While FOXA1 motifs were enriched in both types of loci (Fig. 4F), the AP1 motif was only enriched at anti-cooperative sites (Fig. 4G). AP1 is an important K562 TF that exhibits some pioneer activity [20]. This finding suggests that a protein-protein interaction between FOXA1 and AP1 underlies anti-cooperative behavior at accessible loci.

## Discussion

Given a definition of pioneer activity that is a TF’s ability to bind at inaccessible genomic locations, we suggest that the difference in the average binding affinity between accessible and inaccessible DNA is a quantitative measure of this activity. We measured pioneer activity indices for FOXA1 and HNF4A in K562 cells and showed that HNF4A has stronger pioneer activity in this cell type than FOXA1. However, both TFs showed a range of dox_50_ values across the genome, which demonstrates that a TFs pioneer activity may depend on accessibility, native chromatin marks, and other factors. Some of these differences are explained by the motif content at different locations, suggesting that low affinity interactions at inaccessible binding sites can be overcome by strong motif content. While our work shows that the pioneering activity of a TF can vary across the genome, what accounts for this variation across sites remains mostly unexplained. DNA accessibility had the largest effect on pioneer activity but only explained 17% of the variance in dox_50_ values. We speculate that much of the remaining variance in dox_50_ values might be explained by interactions with other specific TFs, chromatin remodelers, or with the general transcription machinery that can differ across the genome.

Our work supports the hypothesis that pioneer activity is not a qualitative trait limited to a few TFs, but rather a quantitative property of TFs that manifests differently depending on the TF and the genomic environment [1, 3, 4, 7, 8, 21, 22]. Pioneer activity as a quantitative trait fits with data that show that expression level dictates whether a TF adopts a pioneering or collaborating role [23]. We and others have shown that pioneer activity can be enhanced by increasing TF expression [24] or dampened by decreasing TF expression [3].

In vitro, FOXA1 has higher affinity (a lower K_d_) for naked DNA than HNF4A [21, 25, 26], and yet HNF4A has stronger pioneer activity than FOXA1 in K562 cells. These results demonstrate that even though we may parsimoniously consider pioneer activity as a product of binding energies, it may not be sufficient to solely consider a single TF’s DNA-binding domain and its cognate motif. Inside cells, pioneer activity likely depends on the interactions a TF makes with other TFs and with cofactors. Because these interactions will differ in different cell types, a TF’s pioneering activity is also likely to depend on the cell type in which it is expressed and the co-bound TFs present at certain locations.

At some locations in the genome, an interaction between FOXA1 and AP1 appears to have a dramatic effect on FOXA1 activity. In the presence of AP1 sites, FOXA1 displays anti-cooperative binding dynamics where occupancy decreases at the highest levels of FOXA1 expression. We speculate that at these sites monomers of FOXA1 interact with AP1 to potentiate binding, whereas dimers of FOXA1 cannot cobind with AP1. In this model, high concentrations of FOXA1 favor its dimeric form which accounts for the loss of binding at these sites when FOXA1 is expressed at high levels.

## Conclusion

Regardless of the mechanism underlying anti-cooperative behavior, our results show that pioneer activity can be modified by the interactions a TF makes inside cells. Thus, pioneer activity is contingent on many properties of a TF including its levels, its intrinsic affinity for its motif, the motif content at its targets, and the different interactions it makes with other proteins when bound at different locations. Given these contingencies, we suggest that most TFs will display some degree of pioneer activity and that our pioneer activity index will be a useful metric to quantify it.

## Methods

### Cell lines

We grew K562 cells (ATCC CCL-243, Manassas, VA) in Iscove’s modified Dulbecco serum supplemented with 10% fetal bovine serum, 1% penicillin-streptomycin and 1% non-essential amino acids. For each of our functional assays, we split each line into replicate flasks, treated with doxycycline (dox) (Sigma #D9891-1G), and then waited 24 hours to extract nuclei. We used doses of 0.005μg/ml, 0.05μg/ml, 0.1μg/ml, 0.25μg/ml, 0.5μg/ml, and 5μg/ml for our dox_50_ experiments.

### Cloning, production, and infection of viral vectors

We used FOXA1 and HNF4A K562 clonal lines and lentiviral vectors carrying inducible FOXA1 and HNF4A ORFs as described previously [3].

### Sequencing library preparations and analysis

We prepared sequencing libraries and analyzed the two replicates of CUT&Tag as described previously [3]. In our previous work, we already used ATAC-seq to measure the uninduced (-dox) accessibility in the FOXA1 and HNF4A K562 lines [3]. Because we used the same clones to perform these experiments, we re-used these data as uninduced accessibility. We also had already sequenced CUT&Tag libraries for the 0.5-μg/ml and 0.05-μg/ml doxycycline induction levels and re-used these data as well.

### Binding curve analysis

We first established a set of all possible binding sites for each TF by creating a list of binding sites in the sample with the highest dox induction concentration (5μg/ml). We subset this list into those accessible binding sites (called accessible peak in the -dox uninduced condition) and inaccessible binding sites (absence of called accessible peak). Then we used the multiBigwigSummary from the deepTools suite [27] to count the normalized read intensity at each peak from each induction level. We normalized each induction level to the read intensity at the highest induction level in order to convert read intensity into fraction bound.

With these data we fit a binding curve using SciPy curvefit [28] to the equation (Eq. 1) where dox_50_ is unknown and represents a binding affinity parameter similar to K_d_ and where [dox] is the concentration of dox used to induce TF expression. When we plotted examples of randomly selected genomic sites and examined the binding curves, we noticed that at some sites, binding peaked (fraction bound ≥ 1) prior to the highest concentration. In these cases, the fit line estimated a negative dox_50_. For this reason, we filtered out any site that peaked prior to the sample with the highest dox concentration. We also estimated dox_50_ distributions without this filtering step and found similar distributions (Fig. S2). We have listed all of our filtered FOXA1 and HNF4A accessible and inaccessible binding sites, their coordinates, and their dox50 values in a Additional file 2.

In order to quantify the early peak, or “anti-cooperative” behavior that we observed, we classified a binding site as exhibiting a “saturation binding” modality if only the highest dox concentration had a fraction bound of 1, and then each subsequent lower concentration had a lower fraction bound. We classified a binding site as exhibiting an “anti-cooperative” modality if the site peaked at either the third (0.1 μg/ml) or fourth (0.25 μg/ml) dox concentrations and then declined in each direction.

We calculated reproducibility in three ways. We first showed that the binding signal was reproducible by plotting the RPKM signal from each replicate for each of the concentrations at all of the binding sites collected as described above. We then showed that the lines fit similarly between replicates by both replicates’ binding signal and fit binding curves at many different randomly chosen genomic sites and showing that the lines look similar. And finally, we showed that the distributions of dox_50_s from each replicate were highly overlapping. After showing these, we averaged the dox_50_ from each replicate at each site and used the average value moving forward.

### Motif analysis

To discover or count motifs in binding sites, we extracted the sequence from each CUT&Tag binding peak and then used XSTREME [29] for de novo motif discovery and FIMO [30] for specific motif occurrence counting. We used 1e−3 as a *p*-value threshold and JASPAR [31] PWMs for FOXA1 (MA0148.1), HNF4A (MA0114.2), and AP-1 (MA1141.1). We used these motif counts to subset the FOXA1/HNF4A accessible/inaccessible peaks into those with less than 2 motifs, more than 2 but less than 4, or 4 or more, and then re-ran the analysis (Fig. 2D).

### Chromatin modifications and methylation analysis and modeling

We used previously published datasets of histone ChIP-seq and whole-genome bisulfite sequencing [15] to identify patterns of H3K27Ac, H3K4me1, H3K9me3, and H3K27me3 marks, as well as DNA methylation [19]. We used BEDTools [32] to overlap FOXA1 or HNF4A’s binding sites with chromatin marks. We then used python’s statsmodels to run ANOVA analyses on ordinary least squares linear regressions. Each reported variance is the parameter’s sum of squares contribution divided by the total sum of squares.

The methylation dataset that we used reported the percent of reads at each CpG that were methylated. We converted the hg19 coordinates of our filtered FOXA1 or HNF4A accessible and inaccessible binding sites to GrCh38, overlapped them with methylation data, retained sites that had at least 10 reads of coverage, and then averaged the percent methylation of each CpG in each binding site. This analysis resulted in a list of binding sites that each had both a dox_50_ value and a value representing the average methylation level at each CpG. We then binned binding sites by whether the sites were <33%, >33% but less than 66%, or >66% methylated and plotted distributions of dox_50_s.

## Supplementary Information

Additional file 1: Fig. S1. Reproducibility of binding signal. RPKM signal from each replicate of CUT&Tag data across each TF across each dox induction concentration. Pearson’s R correlation displayed on each graph. Fig. S2. Common saturation behavior binding pattern. 16 examples from different genomic sites showing saturating binding signal as dox induction increases. Signal is first read normalized (RPKM) and then normalized to the signal at the highest concentration. These sites were sampled from FOXA1 accessible binding sites, but are common across accessible and inaccessible HNF4A binding sites as well. Fig. S3. Sample of replicate fit binding curves. RPKM binding signal and fitted lines for each CUT&Tag replicate at 16 representative genomic loci. Fig. S4. Replicate dox50 distributions. Dox50 distributions extracted from fitted lines from each CUT&Tag replicate for each TF for each accessibility state. Fig. S5. Dox50 distributions without filtering out early saturation peaks. Dox50 distributions from all of the FOXA1 accessible binding sites (n = 10,118), FOXA1 inaccessible binding sites (n = 17,644), HNF4A accessible binding sites (n = 16,137), and HNF4A inaccessible binding sites (n = 16,507), without filtering out those peaks where binding signal peaked prior to the 5ug/ml dox sample. Fig. S6. Effect of DNA methylation on dox50 distributions. The average CpG methylation (% methylated reads at CpG) per sequence versus the sequence’s dox50 at FOXA1 accessible (R = 0.217) (A), FOXA1 inaccessible (R = -0.107) (C), HNF4A accessible (R = -0.002) (E), or HNF4A inaccessible (R = -0.126). (G) sites. The dox50 distributions at FOXA1 accessible (B), FOXA1 inaccessible (D), HNF4A accessible (F), or HNF4A inaccessible (H) sites binned by whether the site’s CpGs were <33% methylation, between 33% and 66% methylated, or >66% methylated. Fig. S7. Common “anti-cooperative” binding pattern. 16 examples from different genomic sites showing a pattern of increasing and then decreasing binding signal as dox induction increases. Signal is first read normalized (RPKM) and then normalized to the signal at the highest concentration. These sites were sampled from FOXA1 accessible binding sites.

Additional file 2: Table S1. Dox_50_ values at TFBSs with hg19 coordinates.

Additional file 3. Review history

## Data Availability

All genomic sequencing data have been deposited on Gene Expression Omnibus (GEO) under accession number GSE204726 [33]. We used previously published datasets of histone ChIP-seq and whole-genome bisulfite sequencing [15] to identify patterns of H3K27Ac, H3K4me1, H3K9me3, and H3K27me3 marks, as well as DNA methylation [19].
