# Supplementary material for: A quantitative metric of pioneer activity reveals that HNF4A has stronger in vivo pioneer activity than FOXA1
Source: Genome Biol. 2022 Oct 17;23:221. doi: 10.1186/s13059-022-02792-x (PMC9575205; doi:10.1186/s13059-022-02792-x)
Supplement: Supplementary file 1 — Additional file 1: Fig. S1. Reproducibility of binding signal. RPKM signal from each replicate of CUT&Tag data across each TF across each dox induction concentration. Pearson’s R correlation displayed on each graph. Fig. S2. Common saturation behavior binding pattern. 16 examples from different genomic sites showing saturating binding signal as dox induction increases. Signal is first read normalized (RPKM) and then normalized to the signal at the highest concentration. These sites were sampled from FOXA1 accessible binding sites, but are common across accessible and inaccessible HNF4A binding sites as well. Fig. S3. Sample of replicate fit binding curves. RPKM binding signal and fitted lines for each CUT&Tag replicate at 16 representative genomic loci. Fig. S4. Replicate dox50 distributions. Dox50 distributions extracted from fitted lines from each CUT&Tag replicate for each TF for each accessibility state. Fig. S5. Dox50 distributions without filtering out early saturation peaks. Dox50 distributions from all of the FOXA1 accessible binding sites (n = 10,118), FOXA1 inaccessible binding sites (n = 17,644), HNF4A accessible binding sites (n = 16,137), and HNF4A inaccessible binding sites (n = 16,507), without filtering out those peaks where binding signal peaked prior to the 5ug/ml dox sample. Fig. S6. Effect of DNA methylation on dox50 distributions. The average CpG methylation (% methylated reads at CpG) per sequence versus the sequence’s dox50 at FOXA1 accessible (R = 0.217) (A), FOXA1 inaccessible (R = -0.107) (C), HNF4A accessible (R = -0.002) (E), or HNF4A inaccessible (R = -0.126). (G) sites. The dox50 distributions at FOXA1 accessible (B), FOXA1 inaccessible (D), HNF4A accessible (F), or HNF4A inaccessible (H) sites binned by whether the site’s CpGs were <33% methylation, between 33% and 66% methylated, or >66% methylated. Fig. S7. Common “anti-cooperative” binding pattern. 16 examples from different genomic sites showing a pattern of increasing and then dec [file 13059_2022_2792_MOESM1_ESM.docx]

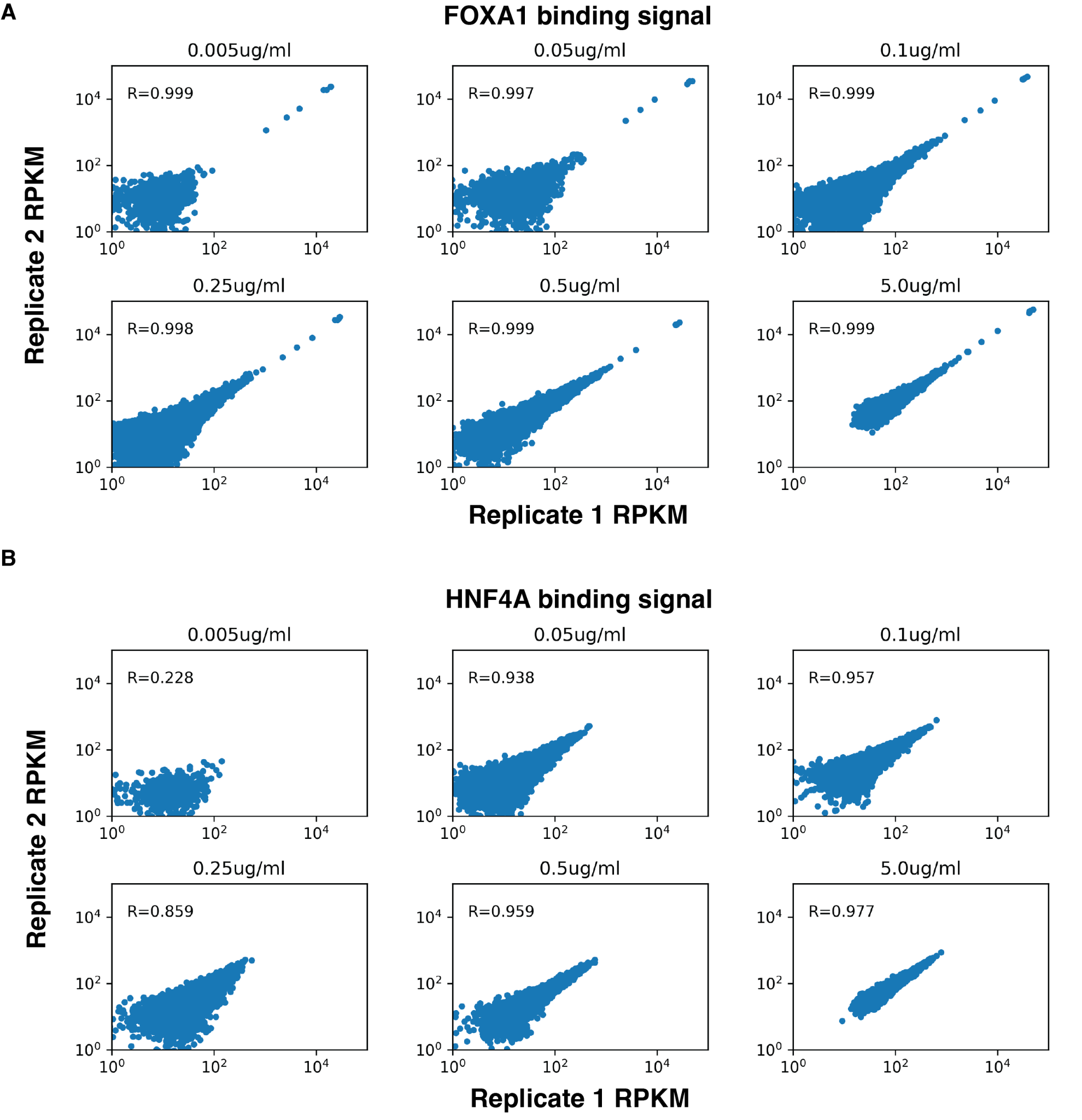


**Fig. S1. Reproducibility of binding signal.** RPKM signal from each replicate of CUT&Tag data across each TF across each dox induction concentration. Pearson’s R correlation displayed on each graph.

**
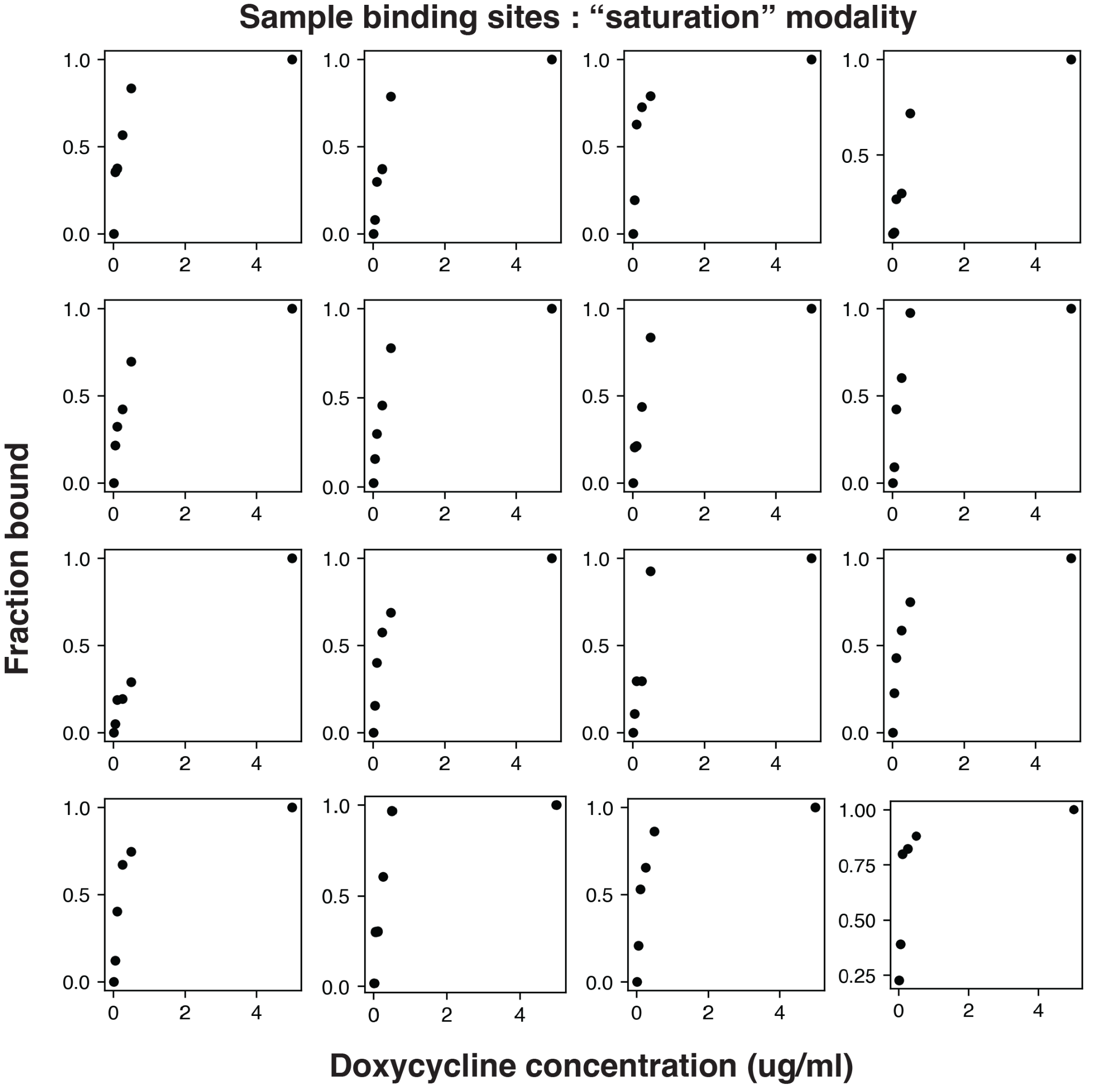
**

**Fig. S2. Common saturation behavior binding pattern.** 16 examples from different genomic sites showing saturating binding signal as dox induction increases. Signal is first read normalized (RPKM) and then normalized to the signal at the highest concentration. These sites were sampled from FOXA1 accessible binding sites, but are common across accessible and inaccessible HNF4A binding sites as well.

**
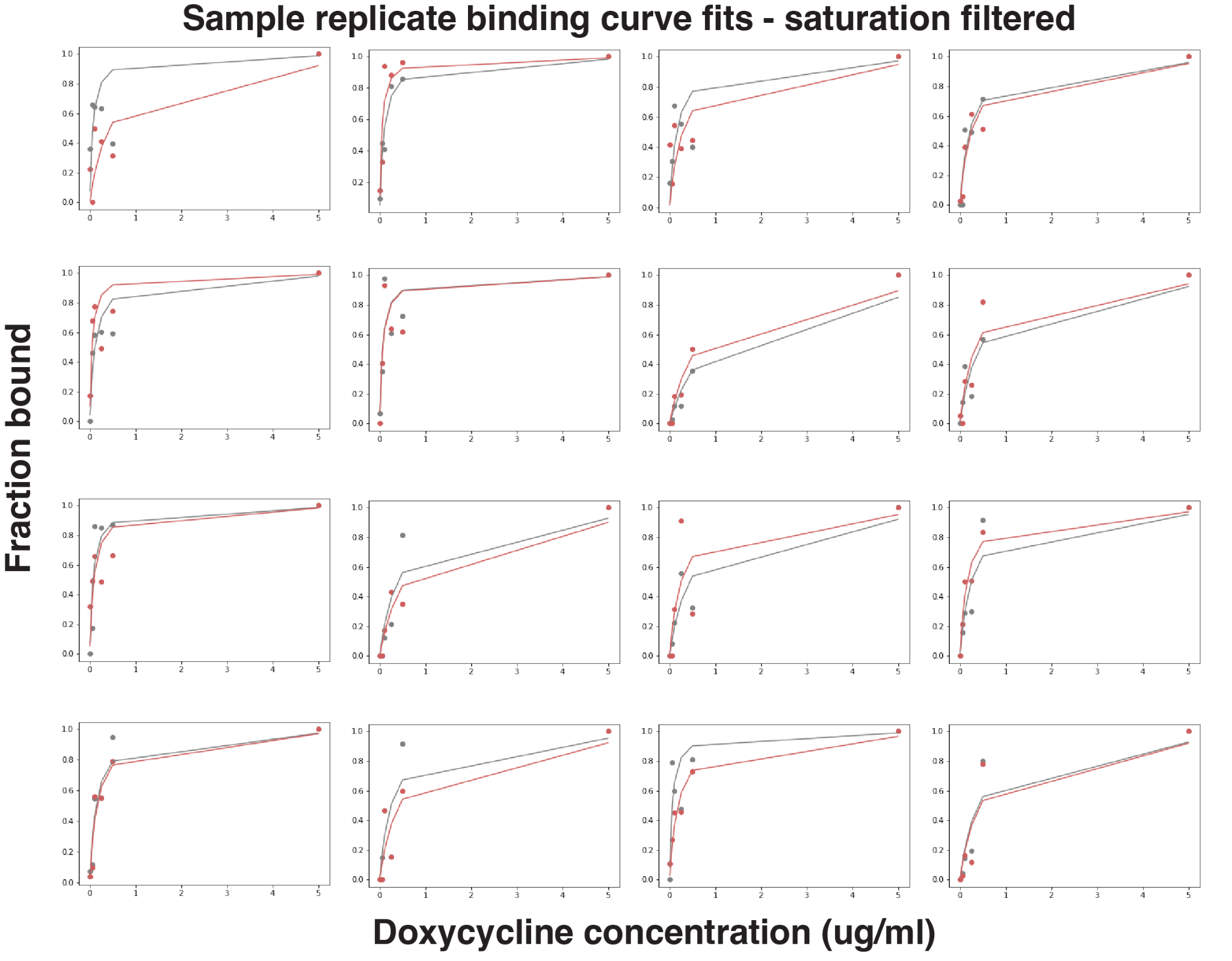
**

**Fig. S3. Sample of replicate fit binding curves.** RPKM binding signal and fitted lines for each CUT&Tag replicate at 16 representative genomic loci

**
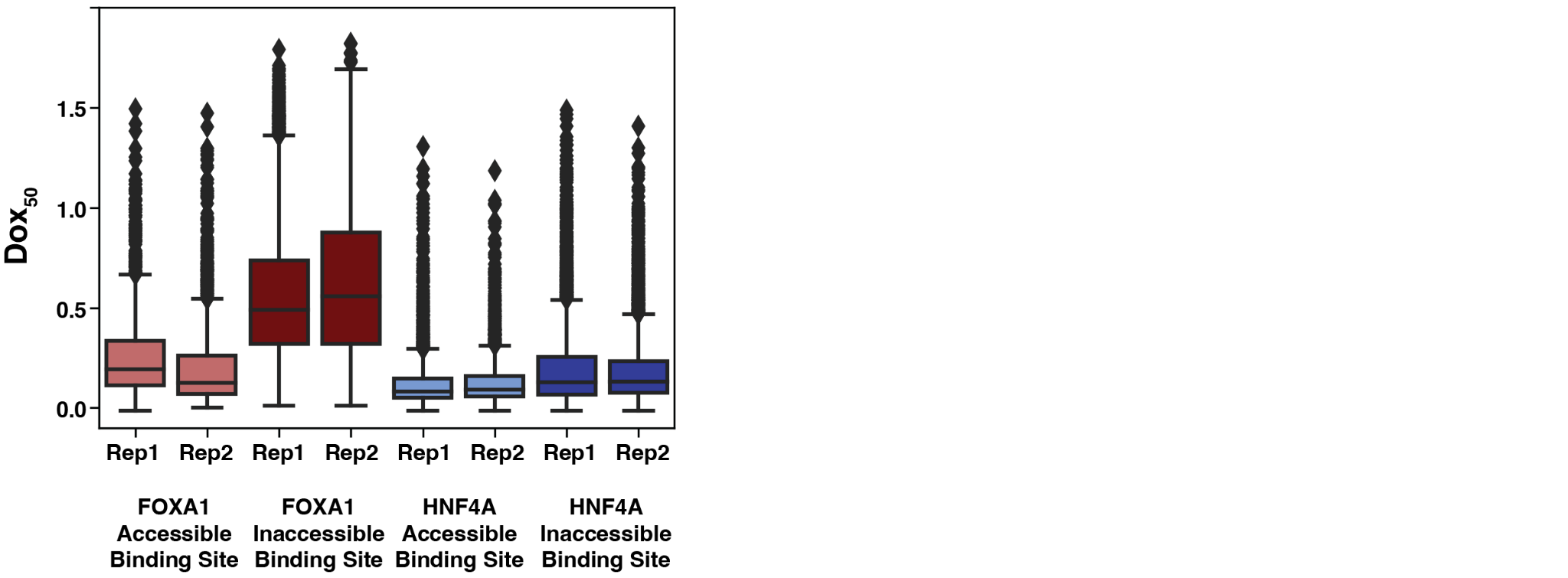
**

**Fig. S4. Replicate dox_50_ distributions.** Dox_50_ distributions extracted from fitted lines from each CUT&Tag replicate for each TF for each accessibility state.

**
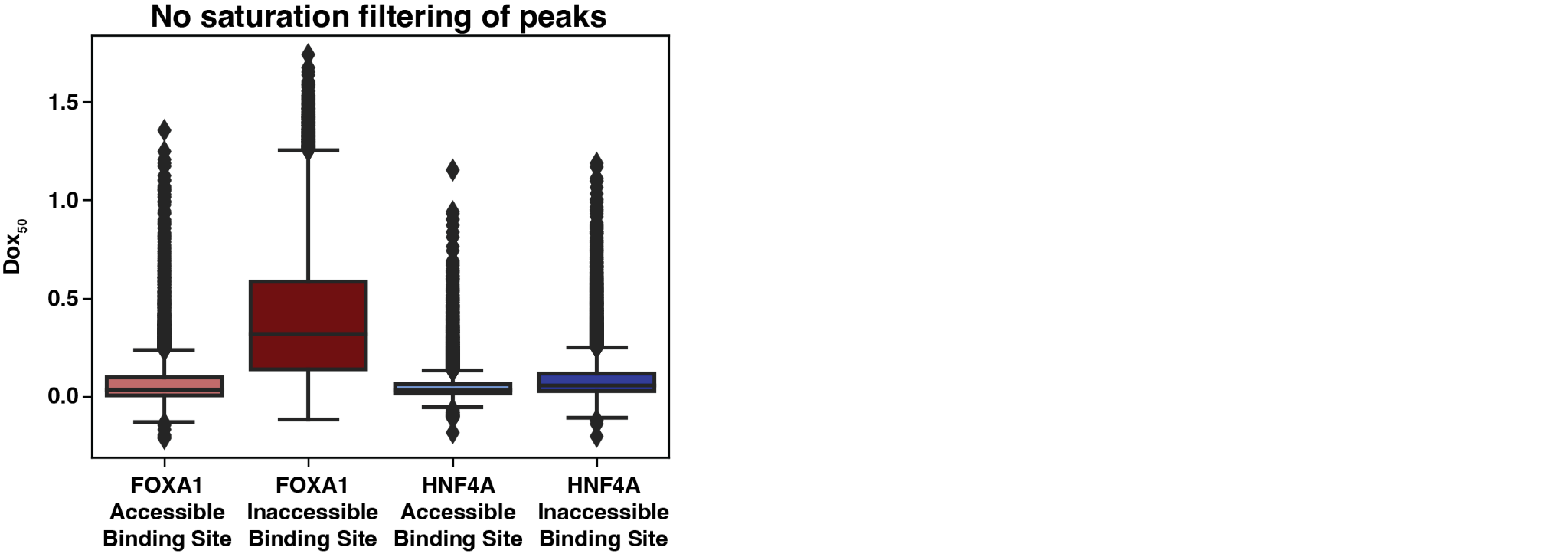

Fig. S5. Dox_50_ distributions without filtering out early saturation peaks.** Dox_50_ distributions from all of the FOXA1 accessible binding sites (*n* = 10,118), FOXA1 inaccessible binding sites (*n* = 17,644), HNF4A accessible binding sites (*n* = 16,137), and HNF4A inaccessible binding sites (*n* = 16,507), without filtering out those peaks where binding signal peaked prior to the 5ug/ml dox sample.


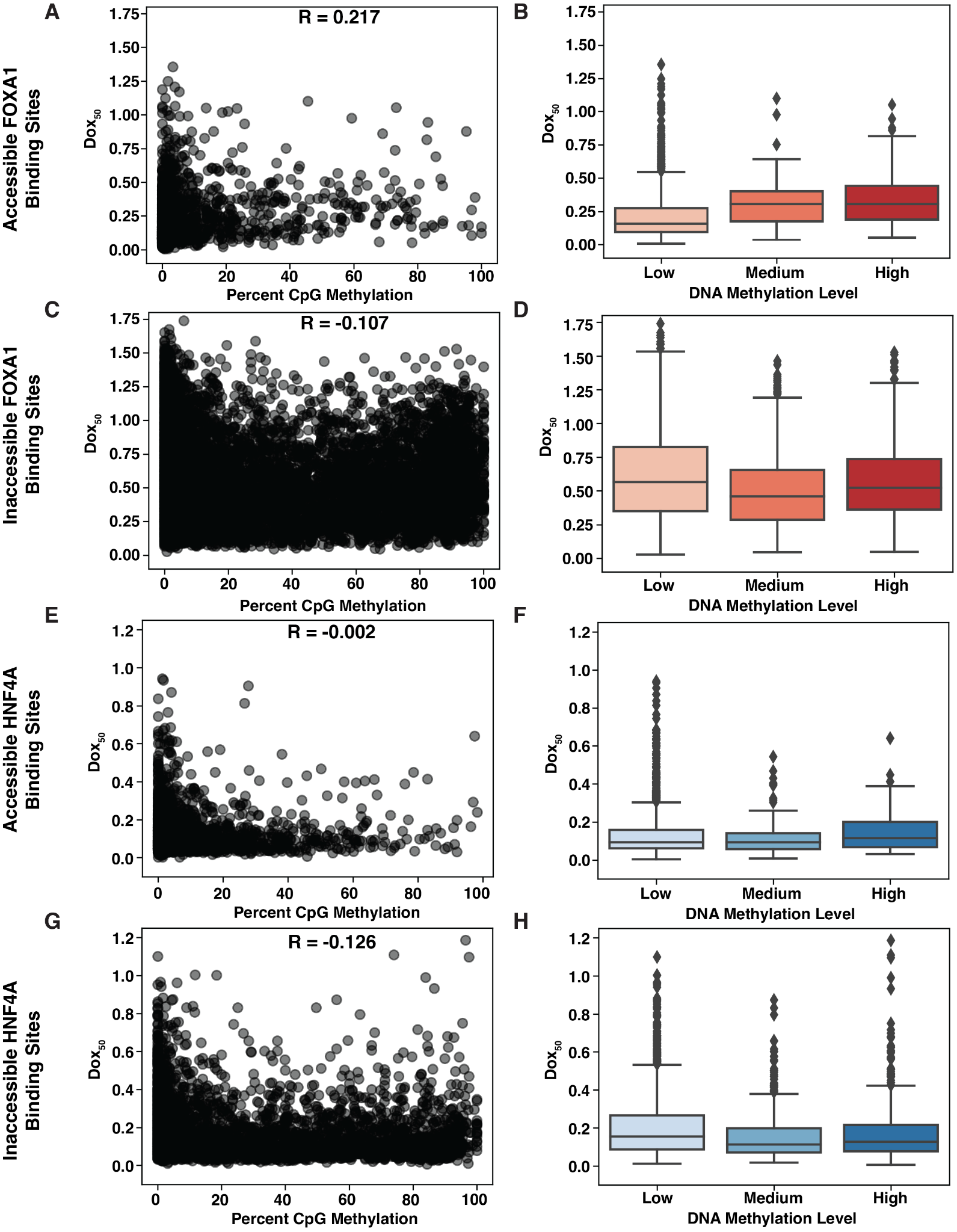


**Fig. S6. Effect of DNA methylation on dox_50_ distributions.** The average CpG methylation (% methylated reads at CpG) per sequence versus the sequence’s dox_50_ at FOXA1 accessible (R = 0.217) **(A)**, FOXA1 inaccessible (R = -0.107) **(C)**, HNF4A accessible (R = -0.002) **(E)**, or HNF4A inaccessible (R = -0.126). **(G)** sites. The dox50 distributions at FOXA1 accessible **(B)**, FOXA1 inaccessible **(D)**, HNF4A accessible (**F)**, or HNF4A inaccessible **(H)** sites binned by whether the site’s CpGs were <33% methylation, between 33% and 66% methylated, or >66% methylated.

**
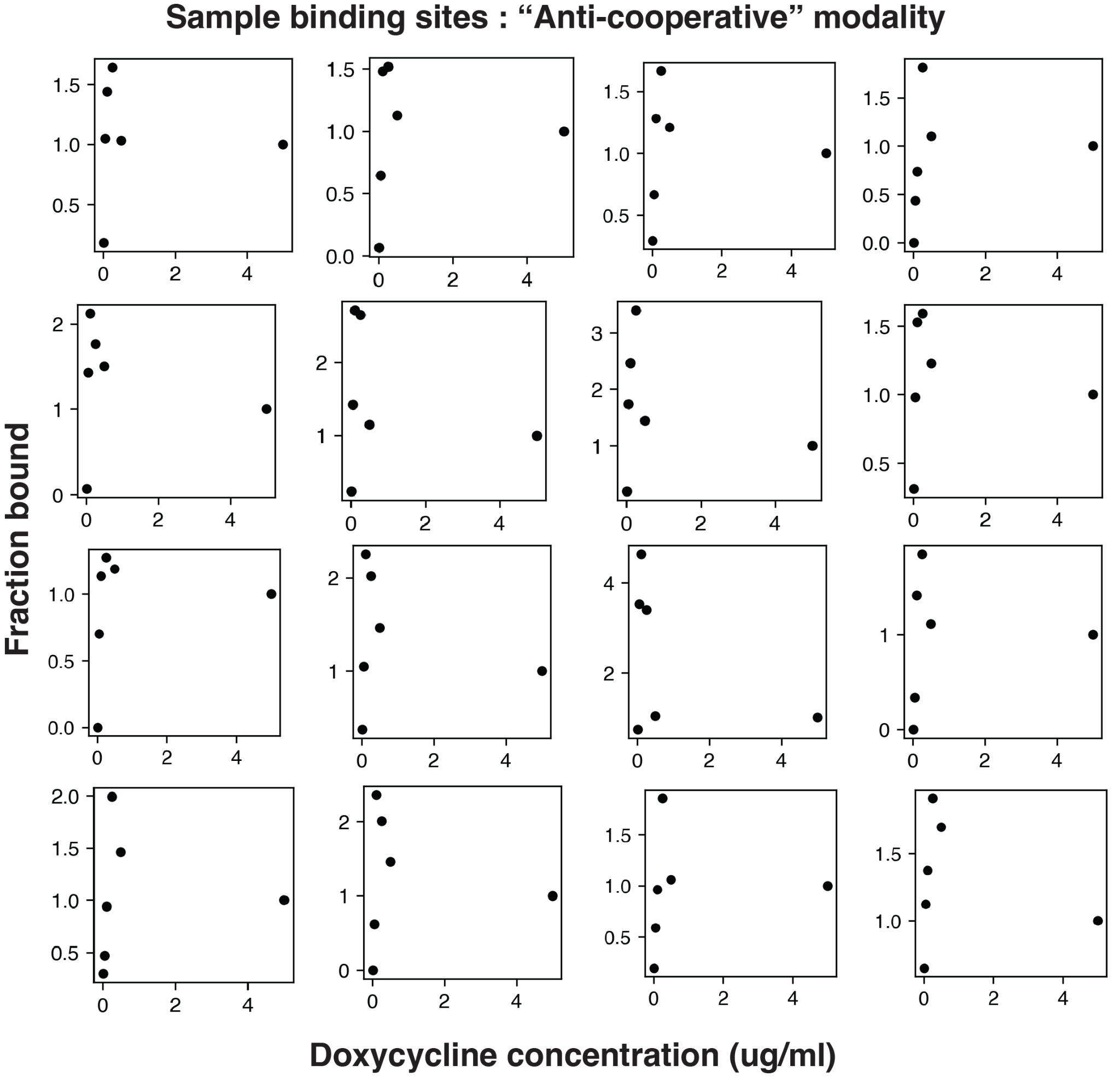
**

**Fig. S7. Common “anti-cooperative'' binding pattern.** 16 examples from different genomic sites showing a pattern of increasing and then decreasing binding signal as dox induction increases. Signal is first read normalized (RPKM) and then normalized to the signal at the highest concentration. These sites were sampled from FOXA1 accessible binding sites.
