# Supplementary material for: A quantitative metric of pioneer activity reveals that HNF4A has stronger in vivo pioneer activity than FOXA1
Source: Genome Biol. 2022 Oct 17;23:221. doi: 10.1186/s13059-022-02792-x (PMC9575205; doi:10.1186/s13059-022-02792-x)
Supplement: Supplementary file 3 — Additional file 3. Review history [file 13059_2022_2792_MOESM3_ESM.docx]

Review History

**First round of review**

**Reviewer 1**

**Were you able to assess all statistics in the manuscript, including the appropriateness of statistical tests used?** There are no statistics in the manuscript.

**Comments to author:**

The mechanism by which TFs (T factors) invade closed chromatin and establish new expression programs is central to an understanding of gene regulation and cell development programs. A widely accepted model, first proposed by Zaret and colleagues, argues that a special set of TFs, called pioneer factors, are responsible for this activity. Other factors are incapable of providing this function, but must follow chromatin opening by the pioneers.

Others have suggested that the pioneering activity is not limited to the special set, but can be achieved by many TFs when targets are in an appropriate chromatin conformation. This remains an important debate in the molecular biology community.

Hansen et al. recently reported [Hansen, Loell, & Cohen, Elife 11, doi:10.7554/eLife.73358 (2022)] that HNF4A, not considered a pioneer factor, is capable of pioneering activity. In the current manuscript, Hansen and Cohen develop an in vivo methodology for determining the "pioneering strength" of a given factor. By expressing the factors under dox regulation, they vary the concentration of the protein within the cell, and determine a metric for it's pioneering, or chromatin penetration, potential. They find that in the cell system under study, K562 blood cells, a lineage in which neither TF is expressed, HNF4A is in fact manifests stronger pioneer potential than the well described classic pioneer, FoxA1. This finding is in disagreement with the standard pioneer model, and casts doubt on the thesis that only a few TFs are capable of pioneering activity.

Hansen and Cohen's work is elegant and makes an important contribution to the ongoing discussion regarding this critical phenomenon.

The work is technically at a high level, well controlled, and would be difficult to challenge on the basis of experimental design and execution.

I find this paper of great interest to the community and strongly support its publication without revision. I have no significant criticisms, either methodological, or in terms of the conclusions.

I would recommend that the authors include reference to two recent papers that support their arguments [Siersbæk, et al. Cancer Cell 38, 412-423 (2020), and Madsen, et al. Mol Cell Biol 34, 939-954 (2014)].

**Reviewer 2**

**Were you able to assess all statistics in the manuscript, including the appropriateness of statistical tests used?** Yes, and I have assessed the statistics in my report.

**Comments to author:**

It has been observed that some TFs have the ability to bind to nucleosomal-bound DNA and potentiate regulatory DNA. TFs which can penetrate closed chromatin a typically called 'pioneer factors'. The difference in 'pioneering' ability between TFs has largely been believed to be a qualitative trait (either a TF can bind into closed chromatin or it cannot). The rationale behind this view stems from the observations that some TFs preferentially bind nucleosomal DNA (such as FOXA1), while others can only occupy naked DNA. While the 'pioneer factor' concept has dominated the literature for the last 20 years, it is still unclear that only certain TFs can engage in independent binding in nucleosomal DNA and catalyze chromatin remodeling. Alternatively, it has been suggested that the 'pioneering' ability of a TF simply reflects its intranuclear concentration and sequence context (e.g., strength of motif and presence of co-factors).

In this manuscript Hansen and Cohen set out to resolve whether 'pioneering' is a qualitative trait (all-or-nothing) or, rather, is a quantitative trait that is determined by more conventional biochemical parameters. To do this, the authors develop a clever approach to titrate the levels of individual transcription factors in vivo using a dose dependent doxycycline inducible expression system. Applying various levels of dox to cells, followed by measuring global TF occupancy using CUT&Tag enabled the authors to determine the dox50, or the concentration of dox required for half maximal TF occupancy, for thousands of individual sites simultaneously.

The authors propose that the ratio of the average dox50 at accessible and inaccessible chromatin (Δdox50) is a quantitative metric of 'pioneer' activity.

The authors apply this approach to examine the pioneering activity of two TFs: (1) the canonical 'pioneer' factor FOXA1 and (2) HNF4A. Overall the authors show that both TFs show a strong concentration dependent pioneering ability indicating that pioneering ability is not a qualitative, endowed trait. In fact, the authors provide solid evidence that HNF4A has stronger pioneering activity that FOXA1. The authors posit that motif content modulates pioneering activity. Finally, the authors observe binding sites that show 'anti-cooperative' behavior, such that overall TF occupancy decreases at higher doses of dox, which may be attributed to negative interactions between adjacent TFs (motif pairs).

Overall, I find this manuscript extremely interesting from a conceptual standpoint. The dox inducible system is extremely clever, and the analytical system is very intuitive and directly addresses the question at hand. However, I found that manuscript is lacking depth in the analysis and overall presentation. The text was missing some key details explaining the background and methodology and the figures are difficult to understand.

1) Introduction needs more information regarding the dox inducible system.

2) I would recommend switching the order of the 'Definition of "Δdox50" parameter for pioneer activity" section with the following section 'Measurement of Δdox50 for FOXA1 and HNF1A'. It would be much clearer if the dox50 concept was described before the Δdox50 metric.

3) Line 95: "FOXA1 and HNF4A appear to be expressed at similar levels at each dox level as each TF bound to similar numbers of sites in each condition (Fig. 1C)." Fig. 1C show the number of sites vs. [dox] - how is that related solely to expression level? (vs. genome motif prevalence, Kd, amongst other features).

4) Fig 1D: There are no labels specifying the TF which corresponds to each site.

5) Fig S2: Provide genomic coordinates for the sampled sites. In addition, the title contains "saturation modality", a term which is not introduced until the last figure of the manuscript.

6) Lines 120-124: Of FOXA1's 11,557 peaks, 1,930 were in accessible regions and 9,627 were in inaccessible regions (10,120 accessible before filtering, 17,644 inaccessible before filtering). Of HNF4A's 5,940 peaks, 2,135 were in accessible regions and 3,805 were in inaccessible regions (16,137 accessible before filtering, 16,507 after filtering).

What do the 10,120 peaks accessible before filtering refer to? Likewise, for the 17,644 inaccessible before filtering, 16,137 accessible before filtering, and 16,507 after filtering.

7) The term Δdox50 (delta dox50) is confusing. Typically, Δ describes the difference, however, here is it used to describe the ratio. In this case, I would recommend calling the Δdox50, the relative dox50 instead. This would clarify the interpretation of figures (Fig. 2A and 2D).

8) The reference to Fig. 2A should also include Fig. S4). Also, there is no references to Figs. 2B or 2C.

9) Fig 2D (and association section in text): For the FOXA1 accessible sites, the dox50 increases with number of motifs. This is in contrast to the FOXA1 inaccessible sites and the HNF4A sites. The state that "higher motif content correlated with more consistent binding of FOXA1…" While there is an overall decrease in the Δdox50 (table 1), it is because there is a positive dox50 trend for the accessible sites (vs. # motifs) and a negative trend for the inaccessible sites. The authors should comment/dig into this a bit more. Could it be related to the 'anti-cooperative' sites?

10) The analysis in the section "FOXA1 behaves anti-cooperatively at a subset of accessible binding sites" is very confusing. It says that 10,000 peaks were sampled from FOXA1 or HNF4A inaccessible or accessible binding sites to determine the prevalence of 'anti-cooperative' behavior. If I understand correctly, there are 11,557 FOXA1 and 5,940 HNF4A peaks. What is the reason for the sampling and how was it performed?

11) In Figs. 4B and C, why do the bars not add up to 10,000 (number of peaks sampled). Is there another modality group not described in the manuscript?

12) Fig. 4A. Here the x-axis is on the log 10 scale in order to evenly space out the x-values (according to the [dox]). However, in the matching supplementary figure (Fig. S6), the x-axis is on a linear scale. The linear scale makes it hard to distinguish that the middle dox concentration confers the highest occupancy. Overall, I would make all the plots in the paper showing [dox] vs. occupancy on the same scales for consistency.

**Reviewer 3**

**Were you able to assess all statistics in the manuscript, including the appropriateness of statistical tests used?** No, I do not feel adequately qualified to assess the statistics.

**Comments to author:**

Hansen and Cohen, 2022

This manuscript describes a new method to assess the "pioneer" activity of transcription factors (TFs). The method is based on regulated expression of transcription factors in cell types where they are not normally expressed, and comparing the binding of the factors to closed and open chromatin. The manuscript is clearly written and logical, and builds on the authors' earlier work published in eLife. Overall the manuscript represents an important step toward a more quantitative analysis of transcription factor-DNA binding in vivo, and should be published after textual revisions to address the points below.

1. On line 55 the authors claim that computing Kd inside cells is impractical. This may be true for true Kd, but not for apparent Kd, which can be measured for example using fluorescent protein-TF fusions. The method they describe could also be made more quantitative by inserting to the genome high and low affinity binding motifs for the studied TFs.

2. line 55: The method to eliminate absolute concentration of transcription factors is clever, but depends on assumption of linearity of expression as a function of doxycycline concentration. This should be clarified.

3. lines 120-125. The effect of different ratios of initial peaks in open and closed chromatin should be clarified. Does this affect the conclusions? In addition, a more quantitative approach would be helpful. What fraction of different quartiles or deciles of peaks by height are in open vs. closed chromatin?

4. lines 165-170. The authors consider chromatin marks but not CpG methylation. Does CpG methylation affect binding of one of the tested TFs, and could this affect the results?

5. line 210. What is meant by "genetic interaction" here? The interaction appears not to be genetic.

6. line 239. Authors consider general transcription machinery but not co-factors or chromatin remodelers. Could this also influence the variance?

7. In discussion, the authors should frame the work a bit better to biochemical principles. It is fully expected that multiple binding sites have more binding energy, and most of what is described here fits a model where the main determinant of binding is binding energy. The Kd for the TFs may be different, but the effective concentration in the nucleus is also a function of the number of bound sites, which is higher for FOX. This, together with the assumption of linearity of the expression makes it difficult to conclude that the simplest biochemical competitive model is not the most parsimonious.

8. The impact of the work would be increased if the authors would not highlight the concept they describe as "deltaDox50" in the abstract, as Dox refers to an antibiotic and is a bit difficult to understand how it relates to intrinsic activity of a transcription factor. A simpler name that refers to the pioneer factor idea but is more quantitative (pioneer potency etc) would make the work easier to understand and more effectively communicate the main point.

Minor:

The authors should consider discussing work by James Briscoe group studying Sox2 binding when Sox2 is expressed at different levels.

**Authors Response**

**Point-by-point responses to the reviewers’ comments:**

We thank the editors and reviewers for their overall positive response to our work. We also appreciate their thoughtful critiques and helpful suggestions. We have addressed the points raised in review as follows.

Reviewer 1:

We thank you for your kind response.

Reviewer 2:

1. The term Δdox50 (delta dox50) is confusing. Typically, Δ describes the difference, however, here it is used to describe the ratio. In this case, I would recommend calling the Δdox50, the relative dox50 instead. This would clarify the interpretation of figures (Fig. 2A and 2D).

In response to this comment we replaced “Δdox50” with “pioneer activity index.” We also switched the numerator and denominator of the metric to make it more intuitive: the higher the pioneer activity index, the higher the pioneer activity. (See lines 26-46, 95, 159, 341, and throughout.)

2. Introduction needs more information regarding the dox inducible system.

I would recommend switching the order of the 'Definition of "Δdox50" parameter for pioneer activity" section with the following section 'Measurement of Δdox50 for FOXA1 and HNF1A'. It would be much clearer if the dox50 concept was described before the Δdox50 metric.

We have added a paragraph to our Introduction that explains our induction system, how we calculate dox50 measurements for each binding site, and how these dox50 measurements relate to the pioneer activity index (lines 64-77).

3. Fig 2D (and association section in text): For the FOXA1 accessible sites, the dox50 increases with the number of motifs. This is in contrast to the FOXA1 inaccessible sites and the HNF4A sites. The statement that "higher motif content correlated with more consistent binding of FOXA1…" While there is an overall decrease in the Δdox50 (table 1), it is because there is a positive dox50 trend for the accessible sites (vs. # motifs) and a negative trend for the inaccessible sites. The authors should comment/dig into this a bit more. Could it be related to the 'anti-cooperative' sites?

We have clarified throughout the text that the main effect that we see is that motif count positively correlates with affinity (or negatively correlates with dox50 distribution). This means that both TFs can compensate for weak affinity at inaccessible sites when there are more instances of their motifs. The exception is at FOXA1 accessible sites where motif count anti-correlates with affinity. We discuss this phenomenon further in our Results section. (See lines 39, 74, 168, and 245.)

4. Line 95: "FOXA1 and HNF4A appear to be expressed at similar levels at each dox level as each TF bound to similar numbers of sites in each condition (Fig. 1C)." Fig. 1C show the number of sites vs. [dox] - how is that related solely to expression level? (vs. genome motif prevalence, Kd, amongst other features).

We have clarified that while we don’t know if our TFs are expressed at precisely the same level, we observe that both TFs transition from binding hundreds of sites to tens of thousands to sites as we increase dox concentration and that both TFs increase similarly, suggesting that we aren’t operatively exclusively at either end of a TF’s dynamic range (lines 121-124).

5. Fig S2: the title contains "saturation modality", a term which is not introduced until the last figure of the manuscript.

We have now clarified what we mean by “saturation modality,” (lines 126-129).

6. What do the 10,120 peaks accessible before filtering refer to? Likewise, for the 17,644 inaccessible before filtering, 16,137 accessible before filtering, and 16,507 after filtering.

The numbers we listed in parentheses referred to the number of FOXA1 and HNF4A sites that were accessible or inaccessible sites prior to filtering out genomic sites that reached maximum occupancy prior to the highest doxycycline concentration. We comment on how the filtering step removed many accessible binding sites, especially for FOXA1, due to the anti-cooperative binding modality (lines 149-155).

7. The reference to Fig. 2A should also include Fig. S4). Also, there is no references to Figs. 2B or 2C.

We have corrected these errors by adding Fig. S4 to the Fig. 2A reference and adding a line referencing 2B-C (lines 157-159).

8. The analysis in the section "FOXA1 behaves anti-cooperatively at a subset of accessible binding sites" is very confusing. It says that 10,000 peaks were sampled from FOXA1 or HNF4A inaccessible or accessible binding sites to determine the prevalence of 'anti-cooperative' behavior. If I understand correctly, there are 11,557 FOXA1 and 5,940 HNF4A peaks. What is the reason for the sampling and how was it performed?

There were 11,557 FOXA1 and 5,940 HNF4A left after filtering out any site that reached maximum occupancy prior to the highest dox concentration. This filtering step necessarily removed the anti-cooperative sites, because those sites have decreased occupancy at higher TF levels. Therefore for the analysis of anti-cooperative sites, we sampled 10,000 peaks from the total set of un-filtered peaks. We sampled 10,000 from both FOXA1 and HNF4A to quantify how many out of 10,000 followed the anti-cooperative modality for each TF. We have clarified this in the text (lines 217-221).

9. In Figs. 4B and C, why do the bars not add up to 10,000 (number of peaks sampled). Is there another modality group not described in the manuscript?

We drew strict criteria for the saturation behavior (binding signal must increase sequentially until maximum at 5µg/ml) and for the anti-cooperative behavior (binding signal must increase sequentially until peak and then decrease sequentially) such that not every one of the 10,000 sampled binding sites were included within one of these two modalities (lines 221-225).

Reviewer 3:

10. The impact of the work would be increased if the authors would not highlight the concept they describe as "deltaDox50" in the abstract, as Dox refers to an antibiotic and is a bit difficult to understand how it relates to intrinsic activity of a transcription factor. A simpler name that refers to the pioneer factor idea but is more quantitative (pioneer potency etc) would make the work easier to understand and more effectively communicate the main point.

See response Revision 1. We have also removed the technical discussion of dox50 from the Abstract. (See Abstract and lines 64-77).

11. On line 55 the authors claim that computing Kd inside cells is impractical. This may be true for true Kd, but not for apparent Kd, which can be measured for example using fluorescent protein-TF fusions. The method they describe could also be made more quantitative by inserting to the genome high and low affinity binding motifs for the studied TFs.

Line 55: The method to eliminate absolute concentration of transcription factors is clever, but depends on assumption of linearity of expression as a function of doxycycline concentration. This should be clarified.

We have added commentary on the alternatives and their pitfalls to measuring in vivo Kds as well as the assumptions that our method makes (lines 84, 86, and 91).

12. Lines 120-125. The effect of different ratios of initial peaks in open and closed chromatin should be clarified. Does this affect the conclusions? In addition, a more quantitative approach would be helpful. What fraction of different quartiles or deciles of peaks by height are in open vs. closed chromatin?

See response to Revision 6 and lines 149-155.

13. lines 165-170. The authors consider chromatin marks but not CpG methylation. Does CpG methylation affect binding of one of the tested TFs, and could this affect the results?

We compared the average CpG methylation of each sequence with its dox_50_ value and found that at accessible FOXA1 binding sites methylation very minimally inhibited binding. The signal was negligible at other types of binding sites. We included this analysis as an additional supplemental figure. See lines 207-209, 362-369, and Additional File 1: Fig. S6.

14. line 210. What is meant by "genetic interaction" here? The interaction appears not to be genetic.

We have changed this to a “protein-protein interaction” in the text (line 234).

15. line 239. Authors consider general transcription machinery but not co-factors or chromatin remodelers. Could this also influence the variance?

We list co-factors as a potential source of variance in this section and have now added chromatin remodelers as an additional source (line 250).

16. In discussion, the authors should frame the work a bit better to biochemical principles. It is fully expected that multiple binding sites have more binding energy, and most of what is described here fits a model where the main determinant of binding is binding energy. The Kd for the TFs may be different, but the effective concentration in the nucleus is also a function of the number of bound sites, which is higher for FOX. This, together with the assumption of linearity of the expression makes it difficult to conclude that the simplest biochemical competitive model is not the most parsimonious.

We agree that a simple biochemical model where binding energy predicts pioneer activity is most likely. *In vitro* measurements suggest that FoxA1 has higher intrinsic affinity for its sites than Hnf4a, which suggests that the model may need to contain more than just the TF of interest and its motif. It may need to include known co-factors and their motifs. We have further clarified this in our Discussion (lines 261-264).

**Second round of review**

**Reviewer 2**

The authors have addressed all of my concerns/comments.

**Reviewer 3**

The authors have addressed all my criticisms. My only suggestions relate to discussing some literature.

I missed in my original review that HNF4 was shown to be able to open chromatin by Rogerson et al. (<https://pubmed.ncbi.nlm.nih.gov/30962179/>). Also, very recently, one relevant paper was published that suggests that SOX2 expression level affects binding pattern (https://pubmed.ncbi.nlm.nih.gov/35550614/).

The authors may wish to cite and or discuss these.
